# Incidence and risk factors for pressure injuries in patients who have undergone vascular operations: a scoping review

**DOI:** 10.1186/s40001-023-01036-3

**Published:** 2023-02-13

**Authors:** Basil Ahmad, Matthew Rubio-Sefati, Michael Mina Yacob

**Affiliations:** 1 Faculty of Health Sciences, Queen’s University, Kingston, ON Canada; 2 University of Waterloo, Waterloo, ON Canada; 3 Department of Surgery, Queen’s University, Kingston, ON Canada; 4 Kingston Health Science Centre, Queen’s University, 76 Stuart St, Victory 3, Kingston, ON K7L 2V7 Canada

**Keywords:** Pressure injury, Vascular diseases, Vascular surgical procedures

## Abstract

**Background:**

Patients who have undergone vascular operations are thought to be at an increased risk for developing pressure injuries; however, the extent to which pressure injuries occur in this population is not clear. This scoping review sought to summarize what is known about the incidence of pressure injuries, and the risk factors for the development of pressure injuries in patients who have undergone vascular operations.

**Main:**

An initial search identified 2564 articles, and 9 English language studies were included. Results showed that due to study design limitations in the available literature preventing hospital-acquired and present on admission pressure injuries to be distinguished, it is difficult to ascertain the incidence rate of pressure injuries in this population.

**Conclusion:**

Certain vascular procedures were found to be higher risk for the development of pressure injuries such as major amputations and lower extremity bypass surgery. In addition to procedural risk factors, patient factors were identified that may be associated with the development of pressure injuries in the vascular population, and these in the authors’ view deserve further exploration. Overall, this scoping review identified an area ripe for future research, the results of which would have implications for wound care in healthcare institutions and at home.

## Background

Pressure injuries (PI), alternatively known as pressure ulcers or decubitus ulcers, are serious adverse events that impose a significant physical and emotional burden on patients [1]. Pressure injuries are mostly preventable, yet they affect a large population worldwide and place an economic burden on the health system [2]. A pressure injury is a defined skin injury localized primarily to pronounced bony regions of the body, such as the head, elbows, heels, and back [3]. It is widely accepted that sustained mechanical loading on soft tissue is involved in the etiology of pressure injuries, where increases in the interstitial pressures within the tissue lead to compromised tissue perfusion, and subsequent local tissue ischemia [4, 5]. Ongoing research is revealing that shear, friction and microclimate also play a significant and complex role in the extrinsic factors related to pressure injury formation [6].

Pressure injuries are classified into stages, based on the severity of injury. The severity of the injury is interpreted by damage to the underlying tissue layers, as reported by Edsberg et al. The staging system used to classify PIs is primarily based on the physical appearance of the injury and the extent of tissue loss because of pressure and shear. In 2016, the National Pressure Injury Advisory Panel (NPIAP) revised their staging system for pressure injuries, reflecting an increasing understanding of the etiology of pressure injuries. Treatment, reimbursements (where applicable) and prognosis are determined by the PI staging, which is shown in Table 1 [7].

**Table 1:** National Pressure Injury Advisory Panel devised system for staging pressure injuries

Stages	Severity/damage
Stage 1	- Intact, skin - Nonblanchable erythema
Stage 2	- Partial thickness skin loss, exposing dermis - The area is pink/red and moist/blistered - Fat and other deep tissue are not visible
Stage 3	- Full-thickness skin loss - Visible fat and granulation tissue - Slough can be visible - Bone, muscle, cartilage, tendon, ligament, or fascia is not visible
Stage 4	- Full-thickness skin and tissue loss - Bone, muscle, cartilage, tendon, ligament, or fascia are exposed - Epibole often occurs
Unstageable pressure injury	- Obscured, full-thickness skin loss - Tissue loss to an extent that there is difficulty in identifying the involved tissue
Deep tissue pressure injury	- Intact or non-intact skin with localized area of persistent nonblanchable deep red, maroon, purple - Discolorations or epidermal separation

Several studies have noted that vascular surgery patients may be at an increased risk of developing pressure injuries [8, 9]. Total operating time has been associated with the development of pressure injuries [10], and vascular surgery procedures have been known to be long in duration [11, 12]. The fact that vascular surgery patients often require several procedures may predispose them to the development of pressure injuries as there may be additional exposures to the surgical process characteristics that lead to pressure injury formation [13, 14].

Ratliff [8] pointed out the paucity of studies examining pressure injuries in the vascular population. There may be an increased risk in this population, but little is known about the rate at which they occur, especially within different procedures. This affects practice as a fundamental strategy to prevent pressure injuries is for caregivers to manually reposition patients approximately every 2 h [15]. While it can aid in prevention and treatment, manually repositioning patients at risk and visually inspecting skin integrity is time consuming and subjective [15]. There is a clear need to streamline the assessment procedure by quickly identifying those who are ‘not at risk’, to ensure effective use of resources. Information on both patient factors and procedure risk factors would be helpful in this regard.

Therefore, it is essential to review the literature to investigate what is known about risk factors for pressure injuries in patients who are undergoing vascular operations to develop effective risk assessment protocols and preventative programs. To the same point, without understanding the incidence of pressure injuries in this population, prevention and management of these incidents cannot be optimized. The purpose of this scoping review was to determine and summarize what is known about the development of pressure injuries in patients who have undergone vascular operations, specifically regarding incidence and risk factors.

## Methods

This scoping review followed the five-stage protocol established by Arksey and O’Malley [16], the details of which are described as follows.

### Identifying the research question

*What is known from the existing literature about the incidence and risk factors for pressure injuries in patients who have undergone vascular operations?*

### Identifying relevant studies

Peer-reviewed articles were identified by performing a literature search electronically in the OVID Medline, OVID Embase, CINAHL, and Web of Science databases. The search was performed on October 19th, 2022. Foreign languages were excluded due to the time that would have been required to sufficiently translate the relevant articles, although this means that relevant articles may have been missed. Multiple terms were used to search for articles, including: Vascular Surgery or Axillofemoral Bypass Grafting or Embolectom* or Endarterectom* or Angioplast* or Limb Salvage or Thrombectom* or Vascular Grafting or amput*. These terms were combined with terms such as pressure sore* or pressure injur* or bed sore* or bedsore* or pressure ulcer* or decubitus*. No further keyword restrictions were applied to maintain breadth of the search. The search strategies can be found in Appendix A.

### Study selection

There was a two-step process employed in the study selection. First, retrieved titles and abstracts were independently screened by two researchers according to predetermined criteria. Next, full-text screening was conducted on the articles remaining.

#### Inclusion criteria

Studies were included if they examined a vascular surgery population AND examined the occurrence of pressure injuries OR risk factors for the development of pressure injuries in the population.

#### Exclusion criteria

Findings were excluded if: they examined a surgical population other than vascular; if there was no outcome of interest; or if the full text was unable to be retrieved. Letters, comments, and correspondence forms of publication were also excluded, as well as papers published in a language other than English.

### Charting the data

To address the research question, relevant data were extracted and charted into Table 2 according to the following categories: author(s)/year of study, type of procedure, study design, study sample, relevant data, and results related to the scoping review question. A separate Table 3 included columns risk factors, and studies supported to better present risk factor data.

**Table 2:** Summary of scoping review articles

Author (year of study)	Type of procedure	Study design	Study sample	Relevant data	Result
Ratliff (2020) [8]	Lower limb amputation	Cross-sectional research design	46 patients with peripheral arterial disease. All were vascular surgery patients who were discharged from a level one trauma center from 2016 to 2017	Frequency of pressure injury	Frequency of 37%
Mehaffey et al. (2017) [13]	Open or endovascular carotid repair, open abdominal aortic aneurysm repair, femoral artery to distal vessel revascularization, endovascular peripheral arterial stenting or above and/or below knee amputation	Using the 2009 National Inpatient Survey, all adult patients who underwent vascular surgery were selected	All adult patients who underwent vascular surgery in 2009 in the USA (*n* = 538, 808)	Frequency of pressure injury, risk factors	3.04% percent overall
Thomson et al. (2009) [23]	Elective open abdominal aortic aneurysm repair	National Vascular Database of the Vascular Society of New Zealand was used to conduct retrospective analysis	1549 patients who underwent open abdominal aortic aneurysm repair from 1 January 1994 to 31 December 2005 in all hospitals performing vascular surgery in New Zealand	Incidence, risk factors	0.1 percent of patients developed pressure injury
Spittle et al. (2001) [20]	Lower leg amputations	A retrospective survey of medical and nursing records of all patients	122 patients who had an amputation involving the lower limb in Hereford between January 1995 and December 1998(*n* = 62 for major amputation, *n* = 60 for minor amputation)	Incidence, risk factors	After major amputations, incidence of pressure injury was 55%. After minor amputations, 20%. See Table 4 for risk factors
Schultz et al. (1999) [9]	Vascular surgery	Prospective, randomized design, comparing standard surgical care to new intraoperative mattress overlay	29 vascular surgery patients were included, with 19 using the experimental overlay, and 10 using the standard surgical care	Incidence	45% of patients developed a pressure injury
Aronovitch (1999) [17]	Vascular surgery	1523 packets were mailed to Wound Ostomy, and Continence Nursing Society members who identified their work setting as hospital-based acute care. Each member was asked to collect data on patients who underwent surgery lasting 3 h or longer on Monday through Thursday of a particular week	110 vascular surgery patients met the outlined criteria	Incidence	17.2% of patients developed a pressure injury
Shah et al. (2015) [18]	Open abdominal aortic aneurysm repair, carotid endarterectomy, and lower extremity bypass/femoral endarterectomy	The Nationwide Inpatient Sample (NIS) (2003–2011) was queried to identify never events applicable to vascular surgery patients, including stage 3 and 4 pressure injuries	267,734 patients qualified for inclusion in the study	Frequency of pressure injury, risk factors	Frequency of 0.05%. See Table 3 for risk factors
Corniello et al. (2014) [25]	Vascular surgery	Retrospective medical review	Sample included 849 patients with vascular diseases, who were > 18 years old and were receiving hospital care on a 25-bed vascular surgery step-down unit from November 1, 2008, through December 31, 2009	Risk factors	See Table 3 for risk factors
Aragon-Sanchez (2010) [21]	Below/above the knee amputations	Retrospective analysis	283 patients were included, all of whom underwent major amputation between January 1, 1998, and December 31, 2008, at the General Surgery Department and Diabetic Foot Unit of La Paloma Hospital in Las Palmas de Gran Canaria. 221 patients were diabetic and 62 patients were non-diabetic	Incidence, risk factor	Incidence of 3.5%
Ploeg et al. (2005)[19]	Above knee and below knee amputations	Prospective study	A total of 45 above knee amputations and 77 below knee amputations were performed in 97 patients	Incidence	Incidence of 2.6% in below knee amputations, 15.6% in above knee amputations
Lardenoye et al. (2009) [22]	Amputation, peripheral vascular surgery	Prospective study	204 amputations, and 1370 peripheral vascular surgeries were performed	Incidence	8.8% incidence in amputations, 0.9% in peripheral vascular surgeries
Bath et al. (2018) [24]	Endovascular and open elective abdominal aorta repair	Retrospective study	66,923 patients who underwent abdominal aorta repair between 2009 and 2012	Incidence	In the open group, 49 (0.5%) patients developed a pressure injury and in the endovascular group 117 patients (0.2%) developed a pressure injury, for an overall incidence of 0.25%

**Table 3:** Characteristics reported to be more common in vascular surgical patients with pressure injuries, or reported to be associated with the development of pressure injuries in more than one study in this review (univariate analysis)

Risk factor	Studies supporting
Age	Mehaffey et al. [13], Shah et al. [18]^a^, Spittle et al. [20]
Diabetes	Mehaffey et al. [13], Shah et al. [18]^a^, Spittle et al. [20]
Peripheral vascular disease/atherosclerosis history	Mehaffey et al. [13], Shah et al. [18]^a^, Spittle et al. [20]
Non-elective admission	Mehaffey et al. [13], Shah et al. [18]^a^
Paralysis	Mehaffey et al. [13], Shah et al. [18]^a^
Congestive heart failure	Mehaffey et al. [13], Shah et al. [18]^a^

^a^Only stage 3 and 4 pressure injuries were investigated

### Collating, summarizing, and reporting the results

The initial search identified 5629 articles. The breakdown of articles by electronic database was as follows:

1. OVID Medline (*n* = 324)
2. Embase (*n* = 1024)
3. CINAHL (*n* = 292)
4. Web of Science (*n* = 3989)

Following the literature search as described, the identified research papers were evaluated using Covidence software. Duplicates were first removed, then titles and abstracts were screened. The remaining studies underwent a full-text evaluation to identify only those studies that fulfilled the inclusion criteria.

**Fig. 1:**
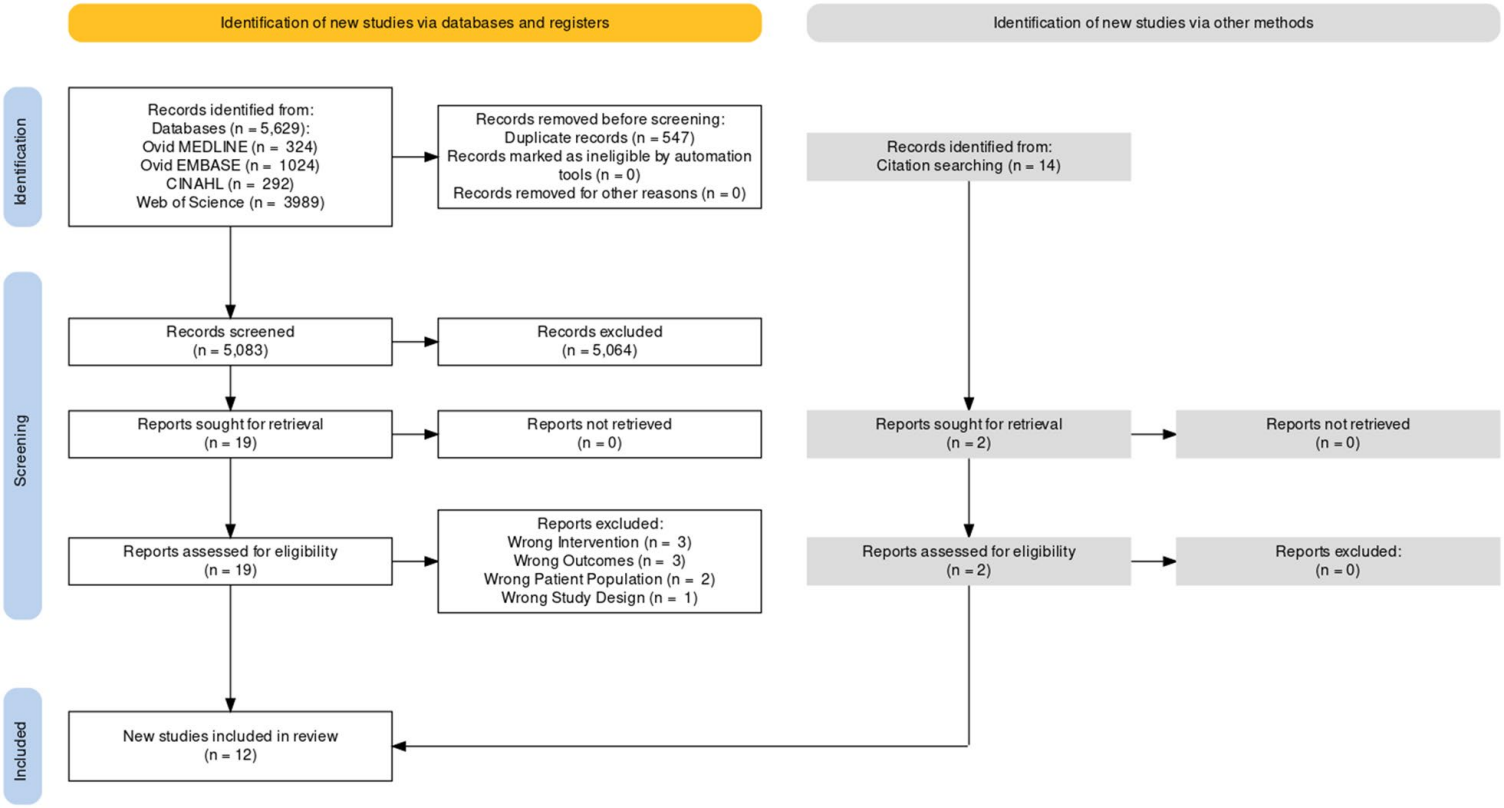
Prisma flow diagram for study selection from databases

In total, 547 duplicate articles were removed, leaving a pool of 5083 articles. This was followed by the exclusion of 5062 articles through title screening and abstract screening, leaving 21 articles to undergo full-text assessment. The search string was found to be quite broad, with the bulk of articles removed during title and abstract screening being on the topic of diabetic, arterial, and venous ulcers. As an example, there were over 500 articles on the topic of diabetic ulcers. During the full-text screening, 12 further articles were excluded, leaving 9 studies for inclusion in this scoping review. Reasons for excluding articles within full-text articles included the presence of irrelevant outcomes, interventions, patient populations, and study designs. Reference list checks were conducted, and this led to 2 additional articles being included. This process is outlined in Fig. 1.

In summary, 12 studies were included in this scoping review. All were primary studies.

## Results

Table 2 shows a summary of the articles included in this scoping review.

### Nationwide frequency study

Mehaffey et al. extracted data from the 2009 Nationwide Inpatient Sample (NIS), which contains data from more than 8 million hospital discharges annually. Of 522,930 patients examined, 15,877 had pressure injuries [13]. This number was also further stratified by procedure type, as shown in Table 4.

**Table 4:** Frequency of pressure injury in 2009 National Inpatient Survey stratified by procedure type

	Number with pressure injury	Number with no pressure injury
Carotid endarterectomy open	261	115 746
Carotid artery stent	261	40 681
Abdominal aneurysm open	463	37 747
Abdominal aneurysm stent	187	38 287
Femoral-distal open	2 519	100 573
Femoral-distal stent	415	29,843
Amputation (above/below knee)	8 508	57 457
Peripheral artery stent	4 974	156 925

It is important to note that it was not possible to determine which pressure injuries were present on admission, and which were hospital-acquired pressure injuries (HAPI). Therefore, they only provide some clue as to the potential range of incidence of hospital-acquired pressure injuries, and the true number is likely lower than the ones provided for all operations. Numbers from this study will be used as a comparison against the incidence rates provided by other studies in this review.

### Unstratified vascular surgery

Aronovitch [17] collected data of surgical patients from 104 acute centers, with a primary objective of determining the presence of intraoperative ulceration. Out of 110 vascular surgery patients, 19 developed a pressure injury, indicating an incidence rate of 17.2%.

Schultz et al. [9] conducted an experimental study to identify the etiology of pressure injuries in a surgical sample and to evaluate a special operating room mattress overlay in order to prevent the development of pressure injuries. 13/29 (45%) of vascular surgery patients developed pressure injuries in the study.

**Fig. 2:**
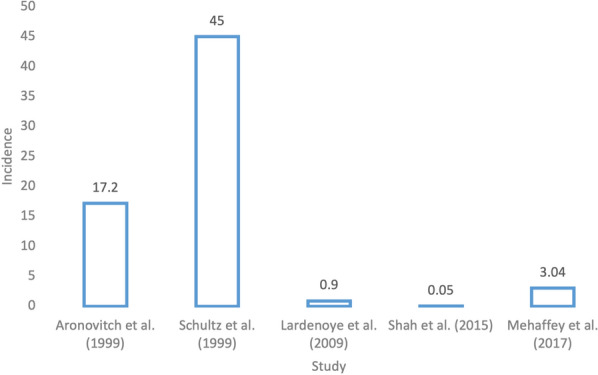
Incidence of pressure injuries in patients after vascular surgery

Shah et al. [18] perused the NIS from 2003 to 2011 to identify stage 3 and stage 4 pressure injuries in vascular surgery patients. The types of procedures included in the study were open abdominal aortic aneurysm (AAA) repair, carotid endarterectomy (CEA), and lower extremity bypass (LEB). In total, 267 734 patient admissions were recorded, with 143 (0.05%) of patients having experienced a stage 3 or 4 pressure injury. It was not able to be determined whether pressure injuries were present on admission or were HAPI. These data are shown in Fig. 2.

Details: Shah et al. [18] (*n* = 267,734) and Mehaffey et al.’s [13] (*n* = 522,930) values are not true incidences as it was not able to be determined whether pressure injuries were present on admission or were hospital-acquired. They are displayed as a reference. In addition, Shah et al. [18] only examined stage 3 and 4 pressure injuries.

### Above/below the knee amputation

Ratliff [8] found that of 46 patients with peripheral arterial disease admitted to a major academic medical center vascular surgery service for below or above the knee amputation, 17 patients (37%) developed a pressure injury upon discharge. There were a total of 19 pressure injuries, indicating that 2 of the 17 patients had 2 pressure injuries. Of the 19 pressure injuries, 10 were present on admission, and 9 were acquired in hospital. Thirteen (68%) of the pressure injuries were on the sacrum. Based on the provided numbers, a minimum of 7/46 patients and a maximum of 9/46 developed a hospital-acquired pressure injury, placing the incidence at 15, 17, or 20%. In Ploeg et al.’s study [19], an incidence of 2.6% was found in below knee amputations, and an incidence of 15.6% was found in above knee amputations, for an overall incidence of 8.0%(*n* = 112).

**Fig. 3:**
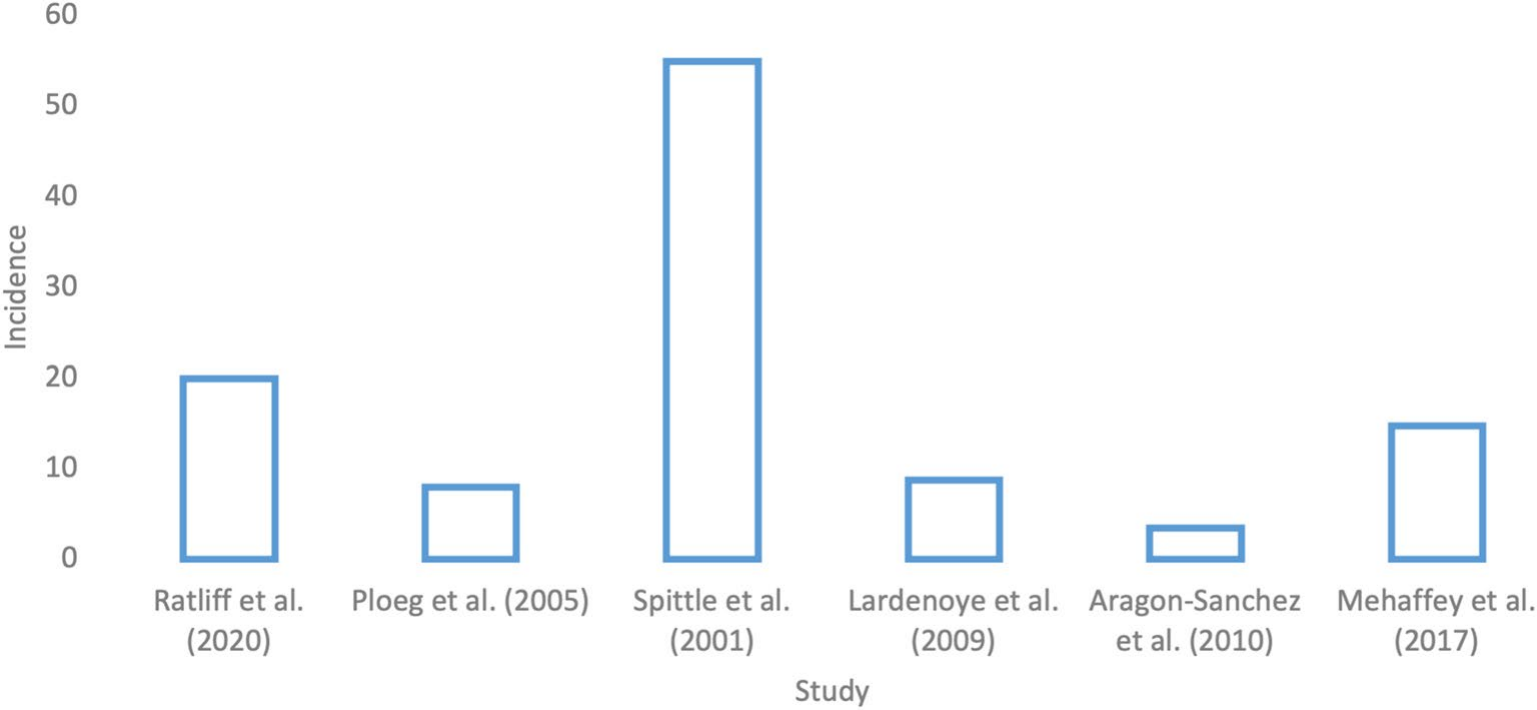
Incidence rate of pressure injuries in studies covering above/below the knee amputations

On the other hand, Spittle et al. [20] conducted a retrospective survey to determine incidence rates for major and minor amputations at a healthcare institution. Major amputations were defined as above-, below-, or through-knee procedures, and minor amputations were those involving the foot [20]. The incidence of pressure injuries after major amputations was found to be 55% [20]. Aragon-Sanchez et al. [21] found an incidence rate of 3.5% (*n* = 283) after major lower extremity amputation in a group of primarily diabetic patients. In Lardenoye et al.’s study [22], 204 amputations were performed, and an incidence rate of 8.8% (*n* = 204) was observed. Further details on studies can be found in Table 2. The aforementioned data are displayed in Fig. 3.

Details: Mehaffey et al.’s [13] (*n* = 57,457) value is not a true incidence as it was not able to be determined whether pressure injuries were present on admission or were hospital-acquired. It is displayed as a reference.

### Abdominal aortic aneurysm repair

Thomson et al. [23] looked at the National Vascular Database of the Vascular Society of New Zealand (NZVASC) to identify all patients who underwent elective open abdominal aortic aneurysm (AAA) repair from 1 January 1994 to 31 December 2005 in all hospitals performing vascular surgery in New Zealand. The goal was to compare complication rates between patients over the age of 80 and those under the age of 80 [23]. Overall, 2 (0.1%) of the 1549 patients selected developed a pressure injury [23].

**Fig. 4:**
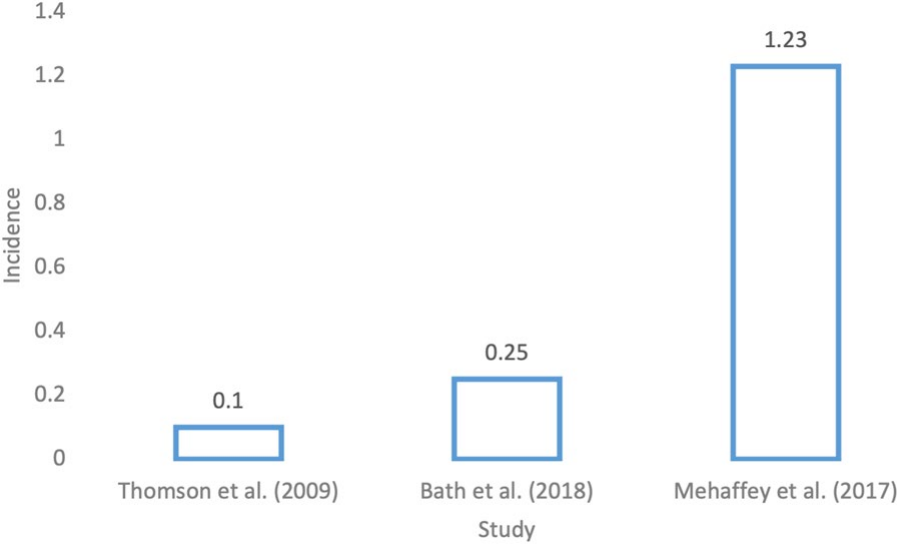
Incidence rate of pressure injuries in studies covering open abdominal aortic aneurysm repair

Bath et al.’s study [24] looked at both elective endovascular and open non-ruptured abdominal aortic aneurysm repairs. A total of 66,923 patients were included in the analysis, 9,315 of which went through open repair, and 57, 608 of which went through endovascular repair. In the open group, 49 (0.5%) patients developed a pressure injury and in the endovascular group, 117 patients (0.2%) developed a pressure injury, for an overall incidence of 0.25%. These numbers are displayed in Fig. 4.

Details: Mehaffey et al.’s [13] (*n* = 37,747) value is not a true incidence as it was not able to be determined whether pressure injuries were present on admission or were hospital-acquired. It is displayed as a reference.

### Risk factors

Four studies examined age as a risk factor [13, 18, 21, 23]. Shah et al.’s [18] study was the exception, finding that age was not associated with the development of pressure injuries in elective open AAA repair.

Diabetes was examined as a risk factor in four studies in this review. Pressure injuries were found to be more common after major amputations in diabetic patients compared to non-diabetic patients by Spittle et al. [20]. Diabetes was also found to be an independent risk factor for the occurrence of stage 3 and 4 pressure injuries in patients undergoing LEB, open AAA repair, or CEA by Shah et al. [18]. Mehaffey et al.’s [13] nationwide study also found that pressure injuries were more common in patients with diabetes. Aragon-Sanchez et al. [21] did not find diabetes to be a risk factor for pressure injury in below/above the knee amputations.

Peripheral vascular disease and atherosclerosis history were found to be associated with an increase in pressure injuries in 2 studies and proposed to be associated in another [13, 18, 20]. Non-elective admission was found to be more common in patients with pressure injuries in two studies in this review.

Corniello et al. [25] conducted a retrospective review of medical records to determine the predictors of HAPI in hospitalized surgical patients with vascular diseases who are treated on a vascular surgery step-down unit. A risk score was created to identify patients at high risk for HAPI after admission to a surgical step-down unit for vascular diseases [25]. This was a multivariate analysis, with factors not necessarily included in Table 3. In these multivariate analyses, nine factors predicted HAPI: lower right ankle brachial index (ABI) and Braden score, an ICU stay, low and high hematocrit values, female sex, non-White race, atherosclerosis history, and higher blood urea nitrogen (BUN) and body mass index (BMI).

## Discussion

This scoping review was designed to report the incidence and risk factors of pressure injuries in patients who are undergoing vascular operations. This scoping review shows that in general, there is a lack of incidence studies for the development of pressure injuries in the vascular surgery population. Due to study design, the structure of electronic databases and digital storage of medical charts, it was difficult for several studies included in this scoping review to ascertain whether pressure injuries were present on admission or were hospital-acquired [8, 13, 18]. Therefore, not a lot is known about true incidence rates for pressure injuries in the vascular surgery population, making it an area ripe for future research.

In 1999, there were a few studies that suggested a high incidence of pressure injuries after vascular surgery, namely 17% and 45% incidence [8, 9]. These studies were not stratified by vascular procedure type and suffered limited sample sizes [8, 9].

Since that time, open AAA repair was found to be a relatively lower risk procedure for the development of pressure injuries by Thomson et al. [23], as 2 (0.1%) of the 1549 patients selected in the study developed a pressure injury after *elective* open AAA repair. This is similar to Bath et al.’s [24] study of 66,923 patients who had elective endovascular and open non-ruptured looked at both elective endovascular and open non-ruptured AAA repairs, only 0.25% of patients developed a pressure injury. However, *non-elective* admission was associated with an increased rate of development of pressure injuries in Mehaffey et al.’s study [13], as well as Shah et al.’s [18] study. Shah et al. [18] postulated that non-elective admissions are more likely to lead to complications due to the urgency of the procedures. This is consistent with the literature, with several studies showing worse outcomes in patients undergoing vascular surgery with non-elective admission [26, 27]. Little is known about the incidence of pressure injuries following open AAA repair in the given context.

Shah et al. [18] recorded additional procedures that appear to relate to the incidence of pressure injuries, including: carotid endarterectomy, and lower extremity bypass surgery. Carotid endarterectomy was not found to be an independent risk factor for the development of pressure injuries, whereas lower extremity bypass surgery was [18]. The authors suggested that this may be because lower extremity bypass surgery is inherently more invasive, has higher rates of systemic complications, and leads to longer length of stay; there is evidence for lower extremity bypass surgery significantly impairing mobility in the postoperative period [28]. Immobility is one of the strongest known risk factors for developing a pressure injury, with the risk of pressure injury increasing as mobility decreases [29]. This study, while accounting for various forms of surgery, limited its scope to only stage 3 and 4 pressure injury types [18]. Therefore, an investigation into the development of stage 1 and 2 pressure injuries in the context of these surgeries would be beneficial, as these also pose a significant burden on patients and the healthcare systems. Furthermore, this investigation would provide clearer insight into occurrence rates as only a small percentage of pressure injuries are stage 3 and 4 pressure injuries [30].

Amputation was found to be consistently reported as a high-risk procedure for the development of pressure injuries with incidence rates in this review ranging up to 55% [20]. However, studies employed small sample sizes, and thus, Mehaffey et al.’s NIS study was referenced. From a sample of 57,457, 14.8% of patients who underwent above or below the knee amputations had pressure injuries [13]. Since hospital-acquired pressure injuries and pressure injuries present on admission could not be differentiated, this number only serves as a benchmark to gain a relative understanding of incidence rates in amputations. Of the 15,877 pressure injuries recorded in this study, 8508 or 53.6% of them occurred in patients who underwent above or below the knee amputation [13]. Amputations stand out as a high-risk procedure even among other vascular surgery procedures. This is not surprising as postoperative immobility is common after amputations [31]. Remaining in the same position for an extended period can lead to interruption of arterial blood supply to a tissue and related blood vessels due to external pressure causes ischemic damage to associated weight bearing tissues [4, 5]. The NPIAP advises that if immobility is the cause of the development of a pressure injury, repositioning and support surfaces for the patient should be addressed [32]. Therefore, it should be ensured that vascular surgical ward nurses and patient families are well educated around these management techniques, especially where amputation is concerned. Lastly, vascular patients who undergo major limb amputation tend to represent the terminal stages of peripheral vascular disease, which inherently, makes them more susceptible to the ischemic mechanism of pressure injury development.

This scoping review identified many risk factors that have been found to be more common in vascular surgical patients with pressure injuries, or that have an association with the development of pressure injuries in this population. Many of those identified fall into commonly cited risk factors for pressure injuries, such as age, diabetes, and vascular disease. To this end, one of the studies included in this review developed a risk score and proved to be predictive of hospital-acquired pressure injuries in a study conducted in a single quaternary care site [25]. This score included factors such as: lower right ABI and Braden score, an ICU stay, low and high hematocrit values, female sex, non-white race, atherosclerosis history, and higher BUN and body mass index (BMI**).** According to the authors, low hematocrit and female sex were risk factors that were not previously documented in other literature [25]. Diabetes was not examined in the study; however, it was noted as a potential risk factor that needs to be studied.

This scale has since been validated in a different setting, including diabetes as a 10th predictive factor [33]. In this subsequent investigation, the 10 factor Vascular HAPI risk score that was created proved to be a valid model to assess risk of HAPI in patients with vascular disease. The Vascular HAPI risk score produced a concordance index of predicted to actual risk of 0.851, and the likelihood of developing an HAPI based on the model was significant (*p* < 0.001). A low Braden score was found to be one of the weaker predictors in the score for hospital-acquired pressure injuries in this population, ranking 9 out of 10 [33]. The Braden score is a widely used scale and incorporates many of the significant factors associated in the development of pressure injuries, such as immobility, friction, and shear. If it is only weakly predictive, this further suggests the need for individualized risk assessment in the vascular surgery population, as the unique profile of vascular patients may be contributing to the frequency of pressure injuries. One suggestion offered by Corniello et al. was based on their finding that low Braden subscale scores in sensory perception and mobility are significant risk factors for the development of pressure injuries [25]. They recommended that Braden subscale factors should be assessed regularly among patients with vascular disease, focusing on the sensory perception and mobility subscales. The Vascular HAPI risk score should be seen as a scale that has the potential to augment the Braden, and further studies are warranted to validate this scale and increase its generalizability in patients of different medical histories.

## Limitations

Our study is limited to several electronic databases and studies in English. Thus, studies published in local journals and those in non-English language were missed. A scoping strategy was used to include all studies on the subject matter at hand, maintaining sufficient breadth and depth of the topic at hand. However, due to this approach, no evaluation was made of the methodological quality of the studies and all levels of evidence were included. The risk factors that lead to the development of pressure injuries are complex. Due to varying study designs and populations, it is difficult to ascertain the relationship that certain variables have with each other.

## Conclusions

Pressure injuries significantly affect quality of life for patients and place a tremendous burden on the healthcare system. Patients who have undergone vascular operations appear to have profiles that uniquely expose them to the risk of pressure injuries, and several risk factors have been identified in the literature, which deserve greater attention in the risk assessment of these patients.

In addition to patient factors, certain procedures such as amputation and lower extremity bypass have been found to be associated with an increased risk for the development of pressure injuries. Amputation appears to be an especially high-risk procedure. Upon discharge, it is important that patients and their families be educated on methods to prevent the occurrence of pressure injuries.

Unfortunately, within the literature, there is a general problem of insufficient data recorded on admission such that it is unknown whether pressure injuries are present on admission or are hospital-acquired. This has led to a landscape of scarce literature when it comes to incidence rates of pressure injuries in patients who have undergone vascular procedures. To remedy this, larger incidence studies are recommended to gain an increased understanding of the rate of pressure injury in the overall vascular surgery population, as well as within specific procedures.

## Data Availability

Not applicable.

## Ovid Medline

exp Vascular Surgical Procedures/ OR (Vascular Surgery or Axillofemoral Bypass Grafting or Embolectom* or Endarterectom* or Angioplast* or Limb Salvage or Thrombectom* or Vascular Grafting or amput*).mp.

## AND

Exp Pressure Ulcer/ OR (pressure sore* or pressure injur* or bed sore* or bedsore* or pressure ulcer*).mp.

## Ovid EMBASE:

exp carotid artery surgery/ or exp microvascular surgery/ or exp endovascular surgery/ or exp aneurysm surgery/ or exp thoracic aortic surgery/ or exp descending aortic surgery/ or exp artery surgery/ or exp vascular surgery/ or exp bypass surgery/ or exp amputation/ OR (Axillofemoral Bypass Grafting or Embolectom* or Endarterectom* or Angioplast* or Limb Salvage or Thrombectom* or Vascular Grafting or amput*).mp.

## AND

Exp decubitus/ OR (pressure sore* or pressure injur* or bed sore* or bedsore* or pressure ulcer*).mp. [mp = title, abstract, heading word, drug trade name, original title, device manufacturer, drug manufacturer, device trade name, keyword heading word, floating subheading word, candidate term word].

WebofScience:

(TS = (vascular surgery)) OR ALL = (Axillofemoral Bypass Grafting or Embolectom* or Endarterectom* or Angioplast* or Limb Salvage or Thrombectom* or Vascular Grafting or amput*).

(TS = (pressure injury)) OR ALL = (pressure sore* or pressure injur* or bed sore* or bedsore* or pressure ulcer* or decubitus*).

## CINAHL

(MH “Vascular Surgery+”) OR Axillofemoral Bypass Grafting orEmbolectom* or Endarterectom*or Angioplast* or Limb Salvage or Thrombectom* or VascularGrafting or amput*

## AND

(MH “Pressure Ulcer+”) OR pressure sore* or pressure injur*or bed sore* or bedsore* or pressure ulcer*
